# Supplementary material for: How does subjective social status at school at the age of 15 affect the risk of depressive symptoms at the ages of 18, 21, and 28? A longitudinal study
Source: PLoS One. 2023 Dec 29;18(12):e0296349. doi: 10.1371/journal.pone.0296349 (PMC10756531; doi:10.1371/journal.pone.0296349)
Supplement: Attachment — Submitted filename: Response to Reviewers.docx [file pone.0296349.s002.docx]

Dear PLOS ONE

Thank you for the valuable response and the opportunity to revise our manuscript. We have gone through the review comments by the reviewer and implemented them in the revised manuscript. Please find the manuscript with tracked changes attached as a separate file. In addition, a clean copy of the revised manuscript is attached. Below you will find replies to each of the questions point-by-point (line numbering refers to the revised version of the manuscript with tracked changes).

1. The authors may want to define the studied time points. Why the time points were chosen? I understand that the study used secondary data, but why the periods were explored still needs to be explained. With well-defined time points, the references could better support the following statements.

Reply:

Thank you for this relevant comment. We have added a short section describing the three life stages; 18 years, 21 years and 28 years, line 106-110

Changes to the text:

“The 3 ages outlined in the study represent important stages in the lives of Danish young people. At 18 years old, the culmination of puberty marks a detachment from the family, though a majority is still living at home. Around the age of 21, most have moved away from home. By the age of 28, most young adults have established their own residences with a steady job and perhaps a family.”

New citation**:** Søndergaard Møller, F. and A. Frølander (2023). Førstegangsfødende er blevet ældre. S. Denmark. www.dst.dk, Statistics Denmark.
https://www.dst.dk/Site/Dst/Udgivelser/nyt/GetAnalyse.aspx?cid=51827

2. Line 64: “The transition from adolescence to adulthood is an important developmental life period in which many physical, psychological as well as social changes occur, and a large number of young people struggle with mental health problems in this particular life phase.” The statement may need to be justified that why changes will bring health problems and consequences for health. Understandably, depression could be the problem that developmental challenges lead to, but the processing or transition needs to be clarified and logicalized.

Reply:

Thank you for bringing this to our attention, we have added the following to the section. Line 70-72

Changes to the text:

“It is well known that young people are particularly susceptible to these changes due to their not yet fully developed and still impressionable minds.”

New citation: World Health Organization. (‎2014)‎. Health for the world's adolescents: a second chance in the second decade: summary. World Health Organization.

3. Line 74-75:＂Social status is often assessed as objective socio-economic status (SES), with classification based on information about income, educational level and/or labor market participation.” The statement is oversimplified with respect to the definition of social status. There are several categorizing methods with their respective concepts. Please provide references to support the categorizing method used in this study.

Reply:

We agree that there are more ways to classify objective social status than the ones used in this study. We have therefore made changes to the text to make this clearer, and we have also added a reference that mention some of these, including those used in this study) as well as a reference that mention typical SES measures in a Danish context. Line 80-82

Changes to the text:

“Social status is often assessed as objective socio-economic status (SES), which can be quantified through various methods (choi YJ et al). In a Danish context, objective socio-economic status is often determined by factors such as income, educational level and/or labor market participation (Poulsen et al.)”

New citation: Choi YJ, Jeong BG, Cho SI, Jung-Choi K, Jang SN, Kang M, Khang YH. [A review on socioeconomic position indicators in health inequality research]. J Prev Med Public Health. 2007 Nov;40(6):475-86. Korean. doi: 10.3961/jpmph.2007.40.6.475. PMID: 18063903.

4. Line 78-80: “Several studies have found low SSS in adulthood to be linked to mental health conditions including depression and depressive symptoms”. Some concern here, the statement is oversimplified how depression or other mental health conditions cause. Please explain the association between SSS and depression/mental conditions to solidify the structure of the manuscript and the following analyses.

Reply:

We acknowledge that the causal relationship is complex and may result from various social and psychological stressors that can be a consequence of low social status. We have added the following to the article and added an additional reference. Line 87-89

Changes to the text:

“For example, a meta-analysis by Quon and McGrath finds that the MacArthur SSS Scale - Youth Version is strongly related to health outcomes related to psychological processes, including depression.”

New citation: Quon, E. C., & McGrath, J. J. (2014). Subjective socioeconomic status and adolescent health: A meta-analysis. Health Psychology, 33(5), 433.

5. Line 80-83: “In young people SSS can be measured in two ways; either as SSS-society which is the self-rated social status the young person assesses his/her family to have compared to other families in the society, or as SSS-school which is the young person's self-rated social status compared to the peers at school.” What is the difference between these two measures? Please explain the meaning and impact of these two measures on adolescents with references to support and rationalize the SSS used in the study.

Reply:

Thank you for pointing this out. The difference between the two concepts is discussed in the line 93-94 and now a reference describing the influence of social class in relation to peers, has been added

Changes to the text:

“Glendinning et al thus found that the mutual influence of peers has a great influence on their self-perception and health behavior.”

New citation: Glendinning A, Hendry L, Shucksmith J. Lifestyle, health and social class in adolescence. Soc Sci Med. 1995 Jul;41(2):235-48. doi: 10.1016/0277-9536(94)00316-l. PMID: 7667685.

6. Line 84-86. “Social interactions are important for young people and young people assess their own status and worth in relation to others. Many hours are spent at school and in Denmark 98% of children are enrolled in either private or primary school.” These sentences are not logical. Please re-organize the sentences.

Reply:

Thanks for pointing this out, the section has now been rewritten. Line 95-99

Changes to the text:

“Social interactions are important for young people as they gauge their own status and value in comparison to their peers. In Denmark, 98% of children attend either private or primary school, spending a substantial portion of their time with their schoolmates. Consequently, comprehending the attributes associated with youth social status requires an examination of the social relations at school.”

7. Line 87-90: “A cross-sectional study by Goodman et al. showed that the average school income, has a positive impact on reducing adolescents' depressive symptoms.” Why school income? It was mentioned that SSS is based on information about income, educational level, and/or labor market participation”. It seems like income is not simply the only factor of SSS. Also, what “school” income? Could the statements and references limit the meaning of SSS?

Reply:

Thank you for this relevant comment. This information should make it clear that the school's financial situation is important for the school context, which may possibly have an impact on how the young person assesses his/her subjective social status in relation to his/her classmates.

We have discussed that this further and agree that this may not be relevant in this study and therefore the sentence has now been removed from the article.

8. The study title is “How does subjective social status at age 15 affect the risk of depressive 4 symptoms in young adulthood? A longitudinal study”; however, “the aim of this study is to investigate the association between SSS-school at age 15 and the risk of developing depressive symptoms at ages 18, 21 and 28.” They don’t seem consistent. To unify the study’s variables, the authors may want to clarify the confusion.

Reply**:**

To create more consistency between the study´s title and aim, the title has now been changed to:

"How does the subjective social status at school at the age of 15 affect the risk of depressive symptoms at the ages of 18, 21, and 28? A longitudinal study”

9. The authors may want to define “15-year-old's subjective social status” as social status in school, with related and supportive references for the present study. However, the definition is unclear, and its meaning and the association with potential mental conditions are unfound in the introduction section, which could confuse the structure of the study.

Reply:

To make the definition more, clear we have added the following to the text, line 111-113

Changes to the text:

“SSS is reliable in predicting physical illness and mental health - including depression, and a study by Lemeshow et al. also shows that SSS among adolescents plays a causal role in shaping health and general wellbeing.”

New citations:

Chen, E., & Paterson, L. Q. (2006). Neighborhood, family, and subjective socioeconomic status: How do they relate to adolescent health? Health Psychology, 25(6), 704.

Lemeshow, A. R., Fisher, L., Goodman, E., Kawachi, I., Berkey, C. S., & Colditz, G. A. (2008). Subjective social status in the school and change in adiposity in female adolescents: Findings from a prospective cohort study. Archives of Pediatrics & Adolescent Medicine, 162(1), 23-28.

10. Why the authors focused on depression in this study? The authors’ motivation, the research background, and the associations between variables were not explained enough. The logic of the study was not recognized in the study.

Reply:

Thank you for the important question. Mental health is declining internationally, and the prevalence of depression is increasing especially among Danish youth, therefore it is of great interest and relevance to investigate factors related to this development. To make this clearer in the text, the following has been added to the text, line 63-67.

Changes to the text:

“Around 15% of Danish adolescents are diagnosed with a mental illness or other developmental disorder before the age of 18, especially children and adolescents with the lowest socioeconomic and social status are at risk. In early adolescence - the period after primary school - young people are particularly vulnerable to developing depression. This may be because, they now stand more on their own two feet and has to make decisions that affect their future life.”

11. Line 121: Since the data was secondary, the flowchart of how the participants were recruited is not proper here.

Reply:

It is correct that the data has already been collected when the study was conducted. However, it was prospectively collected, and we believe it is relevant for the reader to be able to see response rates for the different data collection rounds and the distribution of non-responders and those lost to follow-up as they vary between rounds.

12. Line 133-135: “Each item was a description of a feeling for which the respondent had to indicate the degree to which it applied to him/her. The degree of agreement could be indicated as "Not at all", "A little", "Sometimes" or "A lot".” How many points do these items represent?

Reply:

The score could range from 0 to 3 for each item, summing up to a score between 0-12 as mentioned in line 138. It is now described more in debt in the text, line 152-155.

Changes to the text:

"……the four items were “I was happy”, “I felt like kids I know were not friendly or that they didn’t want to be with me”, “I felt sad” and “It was hard to get started doing things”. The degree of agreement could be indicated from "Not at all", "A little", "Sometimes" or "A lot" equivalent to a score between 0 and 3. ”

13. Line 137: “In this study, participants could score from 0-12 by answering the four questions.” What are the 4 questions? How to do the scaling? Still indicated from "Not at all", "A little", "Sometimes" or "A lot"? Do they scale from 0-3? Please clarify the scaling method.

Reply:

Please see answer to question 12. In addition, the four questions have been added to the text making the scaling method clearer, line 169-173

Changes to the text:

“Each item was a description of a feeling for which the respondent had to indicate the degree to which it applied to him/her, the four items were “I was happy”, “I felt like kids I know were not friendly or that they didn’t want to be with me”, “I felt sad” and “It was hard to get started doing things”. The degree of agreement could be indicated from "Not at all", "A little", "Sometimes" or "A lot" equivalent to a score between 0 and 3. ".

14. Why the SSS-school and SSS-society were categorized and divided into three categories? What is the principle?

Reply:

The reason for the chosen categorization has now been explained in depth. Line 195-198

Changes to the text:

"…allowing for the distinction between two groups of individuals who were distinctly less or more privileged when compared to a middle group. Additionally, this categorization aligns with other studies, making the comparison of results with existing research easier.

New citation**:**

Poulsen PH, Biering K, Winding TN, Aagaard Nohr E, Andersen JH. Influence of childhood family factors on depressive symptoms in adolescence and early adulthood: A Danish longitudinal study. Scand J Public Health. 2020;48(7):715-25.)

15. Line 168-169: Why was the objective socioeconomic status measured by the mean equivalised income and mothers’ educational level? Please provide the principle and references to explain the usage.

Reply:

Thank you for your question. We have edited the text and added relevant references to support the choices. Line 192-199

Changes to the text:

Equivalised household income is chosen as it is a weighted measure based on an OECD modified scale that takes into account household size and composition. Equivalised means that one adult in the household is counted in the calculation by a factor of 1.0, other adults and children over 14 by a factor of 0.5, and children (under 15) by a factor of 0.3. In addition, the annual household income was divided by the weighting factor each year.
In a Danish context, educational level and especially the mother's educational level has been found to have an impact on the future well-being of young people.

New citations:

Hagenaars AJM, Asghar Zaidi M, de Vos K, European Commission, Eurostat (European Commission). Poverty statistics in the late 1980s: research based on micro-data. Luxembourg: Statistical Office of the European Communities; 1994. p. 14–8.

Poulsen PH, Biering K, Winding TN, Aagaard Nohr E, Andersen JH. Influence of childhood family factors on depressive symptoms in adolescence and early adulthood: A Danish longitudinal study. Scand J Public Health. 2020;48(7):715-25.)

16. Line 189: “Bullying” is coming from nowhere and hasn’t been mentioned in the previous statements. Is it related to depression?

Reply:

In the background (line 74-76), bullying, stress and BMI are presented as related to depressive symptoms.

Stress, BMI and bullying were all found to be associated with depressive symptoms and were therefore treated as mediators and confounders respectively in the statistical analysis.

17. Line 194: Why stress and BMI are here?

Reply:

Please see the answer to question 16

18. The “few” versus “many” depressive symptoms seem vague. Authors may want to re-dine the term of the compared set.

Reply:

We agree that the cut-off for depressive symptoms may seem arbitrary. To refine the results and ensure consistency of the association, a sensitivity analysis has been conducted, where the cut-off has been adjusted to the 90.th percentile and the cut-point ≥3. The results of this analysis can be provided upon request. line 350

Changes to the text:

“The results of this analysis can be provided upon request”

19. The multiple logistic regression needs more details while describing the analytic processes.

Reply:

Thank you for your feedback. There has now been made an addition to the text, line 232-235.

Changes to the text:

“Using multiple logistic regression, this study was able to examine the association between an exposure, SSS school, and a dichotomous outcome, depressive symptoms, as well as the influence of a number of co-variates on this association.”

20. In the discussion: “This study is the first to prospectively examine the association between SSS in school and depressive symptoms in adolescence and early adulthood.” The study has processed a great idea by using the data to reveal the phenomenon. However, the research background and literature review must be significantly strengthened to justify and solidify the research concept and structure.

Reply:

Thank you for the feedback. Prior to the study a detailed literature review was done. We have now added the following to the text, line 299-300

Changes to the text:

“Prior to the study, a thorough literature review was conducted. First, a scoping search and a broad search were performed. This was done to identify and narrow down the relevance of the problem.

21. “Several studies have investigated SSS-society as a risk factor of depressive symptoms. This study investigated SSS-school which is a different perspective on the self-perceived social status. The results of our study confirm that SSS-school, may be a more relevant measure of social status among young adolescents than the more societal perspective.” It is a great concept, but no substantial evidence/references support it. Unfortunately, such a problem often happens in this study: Nice perspectives are presented, but they are narrations because of no solid references to support them. Therefore, proper references are strongly encouraged.

Reply:

Thank you for the feedback.

During the process of reviewing your questions, several references have been added to support this.

22. Professional editing is recommended.

Thank you for the comment. The manuscript has been gone through with extra attention to grammar and language

Once again, thank you for your comprehensive review which we believe have led to further improvements of the manuscript.

We look forward to hear from you.

On behalf of the author group,

Best regards,

Marie Kjærgaard Lange, Cand.scient.san
